# [Bis(pyridin-2-ylmeth­yl)amine-κ^3^ *N*,*N*′,*N*′′]tricarbonyl­rhenium(I) bromide hemihydrate

**DOI:** 10.1107/S1600536812019654

**Published:** 2012-05-05

**Authors:** Marietjie Schutte, Theunis J. Muller, Hendrik G. Visser, Andreas Roodt

**Affiliations:** a Department of Chemistry, PO Box 339, University of the Free State, Bloemfontein 9330, South Africa

## Abstract

The title compound, *fac*-[Re(C_12_H_12_N_3_)(CO)_3_]Br·0.5H_2_O, crystallizes with a cationic rhenium(I) unit, a bromide ion and half a water mol­ecule, situated on a twofold rotation axis, in the asymmetric unit. The Re^I^ atom is facially surrounded by three carbonyl ligands and a tridentate bis­(pyridin-2-ylmeth­yl)amine ligand in a distorted octahedral environment. N—H⋯Br, O—H⋯Br, C—H⋯O and C—H⋯Br hydrogen bonds are present in the crystal structure and π–π stacking is also observed [centroid–centroid distances = 3.669 (1) Å and 4.054 (1) Å], giving rise to a three-dimentional network. The mol­ecules pack in a head-to-head fashion along the *ac* plane.

## Related literature

For the synthesis of the *fac*-Re^I^-tricarbonyl synthon, see: Alberto *et al.* (1996 ▶). For a similar structure, see: Banerjee *et al.* (2002 ▶). For related structures, see: Raszeja *et al.* (2011 ▶); Banerjee & Zubieta (2005 ▶); Banerjee *et al.* (2004 ▶, 2006 ▶); Kunz *et al.* (2007 ▶); Wei *et al.* (2006 ▶); Moore *et al.* (2010 ▶).
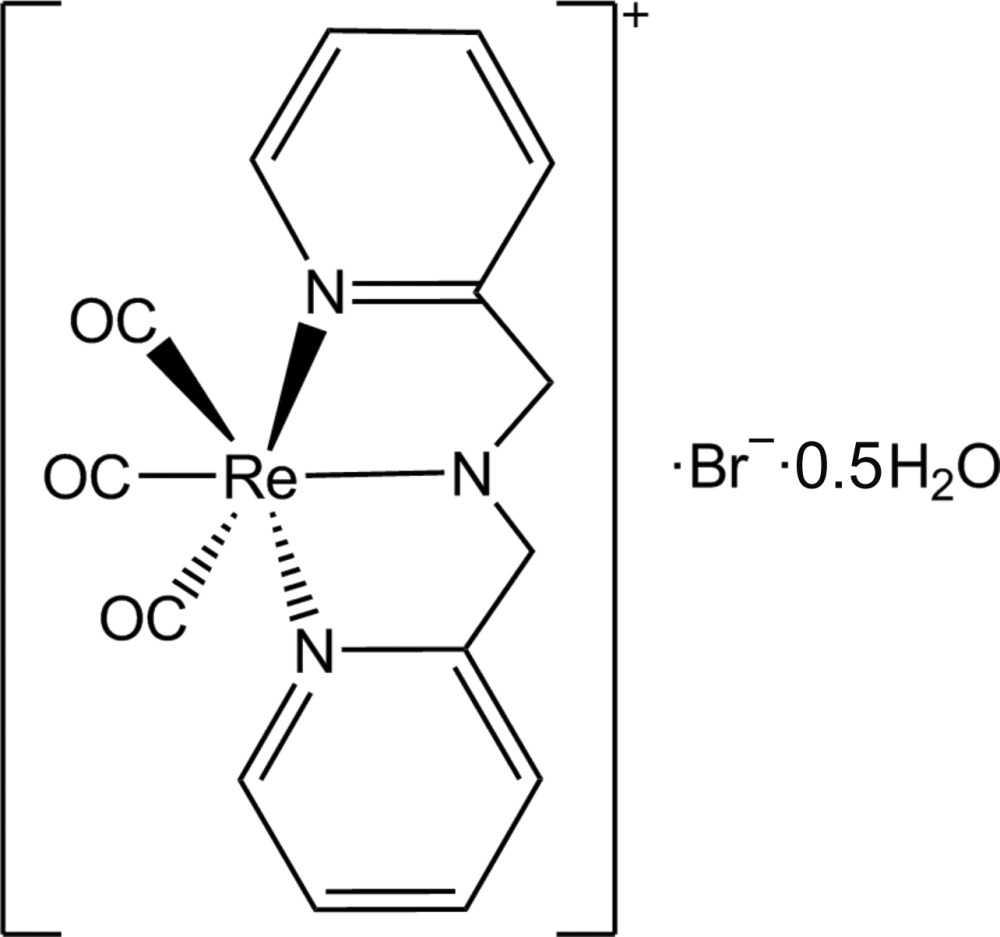


## Experimental

### 

#### Crystal data

[Re(C_12_H_12_N_3_)(CO)_3_]Br·0.5H_2_O
*M* *_r_* = 558.4
Monoclinic, 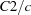

*a* = 21.542 (5) Å
*b* = 11.684 (5) Å
*c* = 15.126 (5) Å
β = 118.172 (5)°
*V* = 3356 (2) Å^3^
*Z* = 8
Mo *K*α radiation
μ = 9.64 mm^−1^
*T* = 100 K
0.34 × 0.12 × 0.09 mm

#### Data collection

Bruker APEXII CCD diffractometer
Absorption correction: multi-scan (*SADABS*; Bruker, 2008 ▶) *T*_min_ = 0.265, *T*_max_ = 0.432
28139 measured reflections
4032 independent reflections
3688 reflections with *I* > 2σ(*I*)
*R*_int_ = 0.031

#### Refinement

*R*[*F* ^2^ > 2σ(*F* ^2^)] = 0.014
*wR*(*F* ^2^) = 0.031
*S* = 1.05
4032 reflections
220 parameters
2 restraints
H atoms treated by a mixture of independent and constrained refinement
Δρ_max_ = 1.14 e Å^−3^
Δρ_min_ = −0.60 e Å^−3^

### 

Data collection: *APEX2* (Bruker, 2008 ▶); cell refinement: *SAINT-Plus* (Bruker, 2008 ▶); data reduction: *SAINT-Plus*; program(s) used to solve structure: *SHELXS97* (Sheldrick, 2008 ▶); program(s) used to refine structure: *SHELXL97* (Sheldrick, 2008 ▶); molecular graphics: *DIAMOND *(Brandenburg & Putz, 2005 ▶); software used to prepare material for publication: *WinGX* (Farrugia, 1999 ▶).

## Supplementary Material

Crystal structure: contains datablock(s) global, I. DOI: 10.1107/S1600536812019654/ru2033sup1.cif

Structure factors: contains datablock(s) I. DOI: 10.1107/S1600536812019654/ru2033Isup2.hkl

Additional supplementary materials:  crystallographic information; 3D view; checkCIF report

## Figures and Tables

**Table 1:** Selected bond lengths (Å)

Re1—C1	1.918 (2)
Re1—C2	1.921 (2)
Re1—C3	1.928 (2)
Re1—N1	2.1819 (19)
Re1—N2	2.1906 (18)
Re1—N3	2.2104 (19)

**Table 2:** Hydrogen-bond geometry (Å, °)

*D*—H⋯*A*	*D*—H	H⋯*A*	*D*⋯*A*	*D*—H⋯*A*
N3—H3⋯Br1	0.85 (2)	2.50 (2)	3.340 (2)	170 (3)
O4—H4*A*⋯Br1	0.94 (2)	2.31 (2)	3.2429 (18)	171 (3)
C11—H11⋯O2^i^	0.93	2.57	3.023 (3)	111
C12—H12⋯O1^ii^	0.93	2.57	3.285 (3)	134
C21—H21⋯O2^i^	0.93	2.56	3.193 (3)	125
C26—H26*A*⋯Br1^iii^	0.97	2.88	3.767 (3)	153
C26—H26*B*⋯O4	0.97	2.31	3.221 (3)	156

Symmetry codes: (i) 
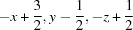
; (ii) 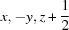; (iii) 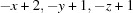.

**Fig. 1.**
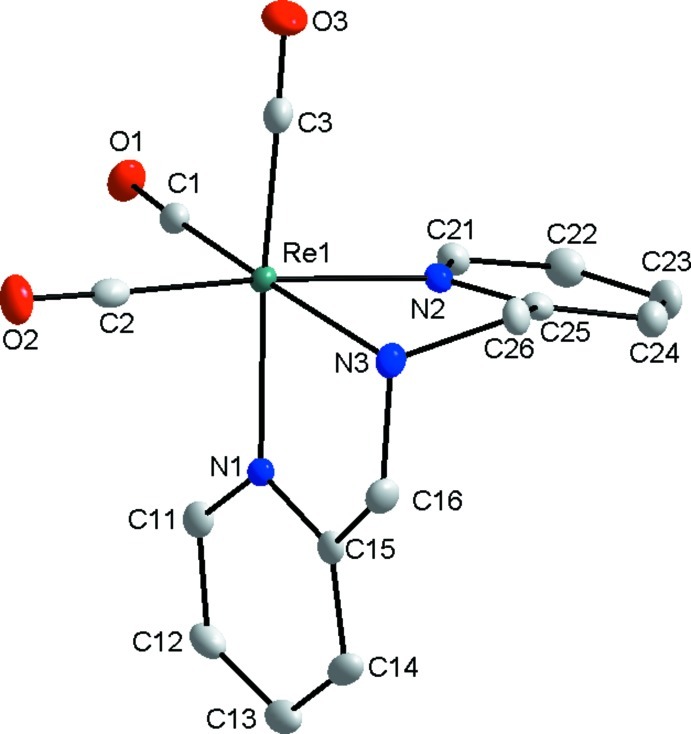
Representation of the title compound, showing the numbering scheme and displacement ellipsoids (50% probability).

**Fig. 2.**
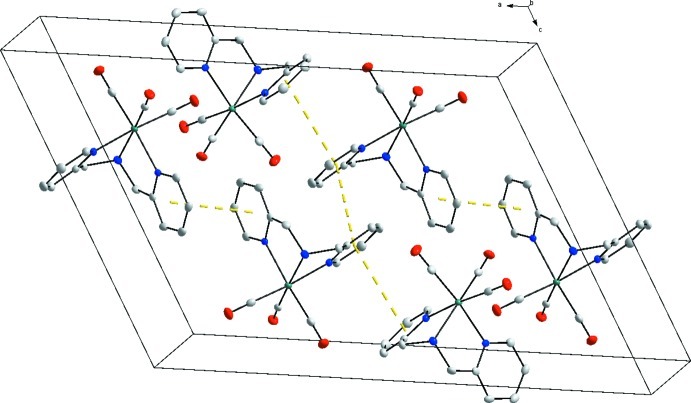
Packing of the title compound in the unit cell and the observed π-π stacking in the crystal structure, indicated by dashed lines (hydrogen atoms omitted for clarity).

## supplementary crystallographic information

### Comment

*N,N*-Bis(2-pyridylmethyl)_2_aminetricarbonylrhenium(I)bromidehydrate crystallized in the monoclinc spacegroup with the cationic *fac*- [Re(CO)_3_({2-pyridyl-CH_2_}_2_NH)], bromide anion and half a water molecule in the assymetric unit. The tridentate ligand, *N,N*-(2-pyridylmethyl)_2_amine, coordinate facially to the Re^I^ core and the other three positions are occupied by carbonyl ligands. The oxygen atom in the water molecule occupies a special position on a mirror plane (Wyckoff position 4*e*, site symmetry 2). Seven hydrogen bonds (N—H···Br, O—H···Br, C—H···O, C—H···Br) are observed in the crystal structure. Some weak π–π stacking, with a centroid-to-centroid distance of 3.669 (1) Å and 4.054 (1) Å, is also observed between the different pyridine rings of the ligand system. These interactions complete a three dimensional polymericnetwork formed between the Re^I^ units. Overall, the bond distances and angles compare well with the similar structure reported by Banerjee *et al.* (2002), *N,N*-bis(2-pyridylmethyl)_2_aminetricarbonylrhenium(I)bromide, that crystallized in the tetragonal *P*4_1_ spacegroup. The three Rhenium to carbonyl distances ranging from 1.918 (2) Å to 1.928 (2) Å compare well to similar structures (Raszeja *et al.* (2011), Banerjee *et al.* (2004), Kunz *et al.* (2007), Wei *et al.* (2006), Banerjee *et al.* (2005), Banerjee *et al.* (2006), Moore *et al.* (2010)) and also to the *N,N*-Bis(2-pyridylmethyl)_2_aminetricarbonylrhenium(I)bromide structure reported by Banerjee *et al.* (2002) of 1.901 (6) Å to 1.926 (7) Å. The Re-amine distance of 2.210 (2) Å and the Re-pyridine distances of 2.182 (2) Å and 2.191 (2) Å are slightly longer than the Re-Amine distance of 2.187 (4) Å and the Re-pyridine distances of 2.177 (5) Å and 2.183 (5) Å reported by Banerjee *et al.* (2002).

### Experimental

[NEt_4_]_2_[Re(CO)_3_Br_3_] (75 mg, 0.097 mmol), as prepared by Alberto *et al.* (1996), was dissolved in 20 ml of water, acidified with HNO_3_ to pH 2.2. Silver nitrate (50 mg, 0.291 mmol) was added to the solution and stirred for 24 h at room temperature. The grey silver bromide precipitate was filtered off, *N,N*-bis(2-pyridylmethyl)amine (19.4 mg, 0.100 mmol) was added to the filtrate and stirred overnight at room temperature. The colourless crystals were grown from the filtrate by slow evaporation.

### Refinement

Aromatic H atoms were positioned geometrically and allowed to ride on their parent atoms, with *U*_iso_(H) = 1.2*U*_eq_(parent) of the parent atom with a C—H distance of 0.93. The methene H atoms were placed in geometrically idealized positions and constrained to ride on its parent atoms with *U*_iso_(H) = 1.2*U*_eq_(C) and at a distance of 0.97 Å. The N– bound H atom was placed from the electron density map.

### Crystal data

**Table:**

[Re(C_12_H_12_N_3_)(CO)_3_]Br·0.5H_2_O	*F*(000) = 2104
*M**_r_* = 558.4	*D*_x_ = 2.21 Mg m^−^^3^
Monoclinic, *C*2/*c*	Mo *K*α radiation, λ = 0.71073 Å
*a* = 21.542 (5) Å	Cell parameters from 9928 reflections
*b* = 11.684 (5) Å	θ = 2.8–28.3°
*c* = 15.126 (5) Å	µ = 9.64 mm^−^^1^
β = 118.172 (5)°	*T* = 100 K
*V* = 3356 (2) Å^3^	Needle, colourless
*Z* = 8	0.34 × 0.12 × 0.09 mm

### Data collection

**Table:**

Bruker APEXII CCD diffractometer	3688 reflections with *I* > 2σ(*I*)
Graphite monochromator	*R*_int_ = 0.031
φ and ω scans	θ_max_ = 28°, θ_min_ = 3.2°
Absorption correction: multi-scan (*SADABS*; Bruker, 2008)	*h* = −28→28
*T*_min_ = 0.265, *T*_max_ = 0.432	*k* = −15→15
28139 measured reflections	*l* = −19→17
4032 independent reflections	

### Refinement

**Table:**

Refinement on *F*^2^	Primary atom site location: structure-invariant direct methods
Least-squares matrix: full	Secondary atom site location: difference Fourier map
*R*[*F*^2^ > 2σ(*F*^2^)] = 0.014	Hydrogen site location: inferred from neighbouring sites
*wR*(*F*^2^) = 0.031	H atoms treated by a mixture of independent and constrained refinement
*S* = 1.05	*w* = 1/[σ^2^(*F*_o_^2^) + (0.0112*P*)^2^ + 4.244*P*] where *P* = (*F*_o_^2^ + 2*F*_c_^2^)/3
4032 reflections	(Δ/σ)_max_ = 0.004
220 parameters	Δρ_max_ = 1.14 e Å^−^^3^
2 restraints	Δρ_min_ = −0.60 e Å^−^^3^

### Special details

**Table:**

Geometry. All s.u.'s (except the s.u. in the dihedral angle between two l.s. planes) are estimated using the full covariance matrix. The cell s.u.'s are taken into account individually in the estimation of s.u.'s in distances, angles and torsion angles; correlations between s.u.'s in cell parameters are only used when they are defined by crystal symmetry. An approximate (isotropic) treatment of cell s.u.'s is used for estimating s.u.'s involving l.s. planes.
Refinement. Refinement of *F*^2^ against ALL reflections. The weighted *R*-factor *wR* and goodness of fit *S* are based on *F*^2^, conventional *R*-factors *R* are based on *F*, with *F* set to zero for negative *F*^2^. The threshold expression of *F*^2^ > 2σ(*F*^2^) is used only for calculating *R*-factors(gt) *etc*. and is not relevant to the choice of reflections for refinement. *R*-factors based on *F*^2^ are statistically about twice as large as those based on *F*, and *R*- factors based on ALL data will be even larger.

### Fractional atomic coordinates and isotropic or equivalent isotropic displacement parameters (Å^2^)

**Table:**

	*x*	*y*	*z*	*U*_iso_*/*U*_eq_	
Re1	0.832406 (4)	0.266160 (7)	0.262052 (6)	0.01016 (3)	
N1	0.82258 (9)	0.22093 (15)	0.39486 (13)	0.0105 (4)	
C3	0.84911 (12)	0.3216 (2)	0.15505 (17)	0.0181 (5)	
N3	0.91084 (10)	0.37861 (16)	0.37892 (14)	0.0130 (4)	
C2	0.74889 (12)	0.35874 (19)	0.21078 (16)	0.0144 (5)	
N2	0.92780 (9)	0.16093 (16)	0.33122 (13)	0.0124 (4)	
C25	0.98776 (12)	0.21485 (19)	0.39603 (16)	0.0134 (4)	
C21	0.92998 (12)	0.04661 (19)	0.31905 (16)	0.0152 (5)	
H21	0.8888	0.0093	0.2746	0.018*	
C22	0.99066 (12)	−0.0171 (2)	0.36981 (18)	0.0204 (5)	
H22	0.9908	−0.0954	0.3586	0.024*	
C24	1.05014 (12)	0.1556 (2)	0.45125 (17)	0.0185 (5)	
H24	1.0906	0.194	0.4966	0.022*	
C23	1.05138 (12)	0.0382 (2)	0.43790 (18)	0.0210 (5)	
H23	1.0927	−0.0029	0.4744	0.025*	
C15	0.86452 (11)	0.27820 (18)	0.48059 (16)	0.0125 (4)	
C11	0.78009 (11)	0.13816 (19)	0.39807 (16)	0.0139 (4)	
H11	0.7496	0.1013	0.3388	0.017*	
C12	0.78005 (12)	0.1058 (2)	0.48615 (17)	0.0164 (5)	
H12	0.7504	0.048	0.486	0.02*	
C16	0.90207 (12)	0.3815 (2)	0.47098 (16)	0.0157 (5)	
H16A	0.8758	0.4496	0.4695	0.019*	
H16B	0.9481	0.3865	0.5294	0.019*	
O2	0.69748 (8)	0.41059 (14)	0.17770 (13)	0.0210 (4)	
O1	0.74405 (8)	0.07008 (14)	0.12519 (12)	0.0212 (4)	
O3	0.85788 (9)	0.35753 (17)	0.09110 (13)	0.0306 (4)	
C1	0.77537 (11)	0.1458 (2)	0.17563 (16)	0.0147 (5)	
C26	0.98272 (11)	0.34246 (19)	0.39980 (17)	0.0151 (5)	
H26A	1.0168	0.3697	0.4656	0.018*	
H26B	0.994	0.3767	0.3507	0.018*	
C14	0.86774 (13)	0.2485 (2)	0.57138 (17)	0.0171 (5)	
H14	0.8983	0.2868	0.6298	0.02*	
C13	0.82505 (12)	0.1613 (2)	0.57449 (17)	0.0185 (5)	
H13	0.8266	0.1404	0.6348	0.022*	
Br1	0.899690 (12)	0.65619 (2)	0.320564 (16)	0.01760 (5)	
O4	1	0.5200 (3)	0.25	0.0578 (10)	
H3	0.9062 (16)	0.4465 (17)	0.356 (2)	0.053 (7)*	
H4A	0.9723 (15)	0.567 (2)	0.268 (2)	0.053 (7)*	

### Atomic displacement parameters (Å^2^)

**Table:**

	*U*^11^	*U*^22^	*U*^33^	*U*^12^	*U*^13^	*U*^23^
Re1	0.00954 (5)	0.01156 (5)	0.00819 (4)	0.00126 (3)	0.00319 (3)	0.00068 (3)
N1	0.0101 (9)	0.0109 (9)	0.0103 (8)	0.0031 (7)	0.0046 (7)	0.0003 (7)
C3	0.0121 (11)	0.0238 (13)	0.0156 (11)	0.0040 (9)	0.0042 (9)	0.0025 (10)
N3	0.0124 (9)	0.0106 (9)	0.0138 (9)	−0.0001 (8)	0.0045 (8)	0.0004 (7)
C2	0.0195 (12)	0.0123 (11)	0.0133 (11)	−0.0026 (9)	0.0093 (9)	0.0000 (9)
N2	0.0114 (9)	0.0143 (10)	0.0125 (9)	0.0016 (7)	0.0063 (7)	0.0016 (7)
C25	0.0133 (11)	0.0162 (12)	0.0126 (10)	0.0005 (9)	0.0077 (9)	0.0004 (8)
C21	0.0143 (11)	0.0174 (12)	0.0154 (11)	0.0015 (9)	0.0082 (9)	−0.0008 (9)
C22	0.0226 (13)	0.0181 (13)	0.0221 (12)	0.0049 (10)	0.0119 (11)	0.0009 (10)
C24	0.0133 (11)	0.0246 (13)	0.0163 (11)	0.0016 (10)	0.0060 (9)	0.0001 (10)
C23	0.0161 (12)	0.0261 (14)	0.0196 (12)	0.0105 (10)	0.0073 (10)	0.0037 (10)
C15	0.0100 (10)	0.0127 (11)	0.0137 (10)	0.0038 (8)	0.0049 (9)	−0.0008 (8)
C11	0.0134 (11)	0.0112 (11)	0.0161 (11)	0.0040 (9)	0.0061 (9)	0.0009 (9)
C12	0.0176 (12)	0.0145 (12)	0.0216 (12)	0.0044 (9)	0.0129 (10)	0.0052 (9)
C16	0.0150 (11)	0.0167 (12)	0.0129 (10)	−0.0014 (9)	0.0044 (9)	−0.0046 (9)
O2	0.0154 (9)	0.0152 (9)	0.0295 (9)	0.0036 (7)	0.0081 (7)	0.0045 (7)
O1	0.0191 (9)	0.0223 (9)	0.0174 (8)	−0.0003 (7)	0.0047 (7)	−0.0083 (7)
O3	0.0287 (10)	0.0461 (12)	0.0224 (9)	0.0075 (9)	0.0166 (8)	0.0134 (9)
C1	0.0124 (11)	0.0191 (12)	0.0120 (10)	0.0050 (9)	0.0053 (9)	0.0023 (9)
C26	0.0100 (11)	0.0169 (12)	0.0171 (11)	−0.0013 (9)	0.0053 (9)	−0.0004 (9)
C14	0.0182 (12)	0.0196 (13)	0.0121 (11)	0.0068 (9)	0.0061 (10)	−0.0014 (9)
C13	0.0197 (12)	0.0205 (13)	0.0179 (12)	0.0096 (10)	0.0110 (10)	0.0071 (9)
Br1	0.01889 (12)	0.01679 (12)	0.01454 (11)	0.00078 (9)	0.00578 (9)	0.00349 (8)
O4	0.091 (3)	0.0332 (19)	0.090 (3)	0	0.076 (2)	0

### Geometric parameters (Å, º)

**Table:**

Re1—C1	1.918 (2)	C22—H22	0.93
Re1—C2	1.921 (2)	C24—C23	1.388 (3)
Re1—C3	1.928 (2)	C24—H24	0.93
Re1—N1	2.1819 (19)	C23—H23	0.93
Re1—N2	2.1906 (18)	C15—C14	1.386 (3)
Re1—N3	2.2104 (19)	C15—C16	1.498 (3)
N1—C11	1.348 (3)	C11—C12	1.385 (3)
N1—C15	1.356 (3)	C11—H11	0.93
C3—O3	1.149 (3)	C12—C13	1.386 (3)
N3—C26	1.488 (3)	C12—H12	0.93
N3—C16	1.491 (3)	C16—H16A	0.97
N3—H3	0.850 (18)	C16—H16B	0.97
C2—O2	1.149 (3)	O1—C1	1.155 (3)
N2—C21	1.352 (3)	C26—H26A	0.97
N2—C25	1.353 (3)	C26—H26B	0.97
C25—C24	1.387 (3)	C14—C13	1.389 (3)
C25—C26	1.498 (3)	C14—H14	0.93
C21—C22	1.380 (3)	C13—H13	0.93
C21—H21	0.93	O4—H4A	0.939 (17)
C22—C23	1.385 (3)		
			
C1—Re1—C2	87.77 (10)	C21—C22—H22	120.8
C1—Re1—C3	89.29 (10)	C23—C22—H22	120.8
C2—Re1—C3	88.99 (9)	C25—C24—C23	119.0 (2)
C1—Re1—N1	98.08 (8)	C25—C24—H24	120.5
C2—Re1—N1	91.64 (8)	C23—C24—H24	120.5
C3—Re1—N1	172.62 (8)	C22—C23—C24	119.6 (2)
C1—Re1—N2	93.94 (8)	C22—C23—H23	120.2
C2—Re1—N2	175.80 (8)	C24—C23—H23	120.2
C3—Re1—N2	94.86 (8)	N1—C15—C14	121.6 (2)
N1—Re1—N2	84.33 (7)	N1—C15—C16	116.82 (19)
C1—Re1—N3	169.31 (8)	C14—C15—C16	121.4 (2)
C2—Re1—N3	101.82 (9)	N1—C11—C12	122.6 (2)
C3—Re1—N3	95.54 (9)	N1—C11—H11	118.7
N1—Re1—N3	77.14 (7)	C12—C11—H11	118.7
N2—Re1—N3	76.18 (7)	C11—C12—C13	118.8 (2)
C11—N1—C15	118.46 (19)	C11—C12—H12	120.6
C11—N1—Re1	124.72 (14)	C13—C12—H12	120.6
C15—N1—Re1	116.78 (14)	N3—C16—C15	112.61 (18)
O3—C3—Re1	177.9 (2)	N3—C16—H16A	109.1
C26—N3—C16	112.72 (17)	C15—C16—H16A	109.1
C26—N3—Re1	109.09 (13)	N3—C16—H16B	109.1
C16—N3—Re1	111.98 (13)	C15—C16—H16B	109.1
C26—N3—H3	105 (2)	H16A—C16—H16B	107.8
C16—N3—H3	108 (2)	O1—C1—Re1	176.56 (19)
Re1—N3—H3	109 (2)	N3—C26—C25	111.18 (18)
O2—C2—Re1	177.3 (2)	N3—C26—H26A	109.4
C21—N2—C25	118.42 (19)	C25—C26—H26A	109.4
C21—N2—Re1	125.07 (15)	N3—C26—H26B	109.4
C25—N2—Re1	116.37 (15)	C25—C26—H26B	109.4
N2—C25—C24	121.8 (2)	H26A—C26—H26B	108
N2—C25—C26	115.44 (19)	C15—C14—C13	119.5 (2)
C24—C25—C26	122.7 (2)	C15—C14—H14	120.2
N2—C21—C22	122.8 (2)	C13—C14—H14	120.2
N2—C21—H21	118.6	C12—C13—C14	118.9 (2)
C22—C21—H21	118.6	C12—C13—H13	120.5
C21—C22—C23	118.4 (2)	C14—C13—H13	120.5

### Hydrogen-bond geometry (Å, º)

**Table:**

*D*—H···*A*	*D*—H	H···*A*	*D*···*A*	*D*—H···*A*
N3—H3···Br1	0.85 (2)	2.50 (2)	3.340 (2)	170 (3)
O4—H4*A*···Br1	0.94 (2)	2.31 (2)	3.2429 (18)	171 (3)
C11—H11···O2^i^	0.93	2.57	3.023 (3)	111
C12—H12···O1^ii^	0.93	2.57	3.285 (3)	134
C21—H21···O2^i^	0.93	2.56	3.193 (3)	125
C26—H26*A*···Br1^iii^	0.97	2.88	3.767 (3)	153
C26—H26*B*···O4	0.97	2.31	3.221 (3)	156

Symmetry codes: (i) −*x*+3/2, *y*−1/2, −*z*+1/2; (ii) *x*, −*y*, *z*+1/2; (iii) −*x*+2, −*y*+1, −*z*+1.

## References

[bb1] Alberto, R., Schibli, R. & Schubiger, P. A. (1996). *Polyhedron*, **15**, 1079–1089.

[bb2] Banerjee, S. R., Babich, J. W. & Zubieta, J. (2004). *Inorg. Chem. Commun.* **7**, 481–484.

[bb3] Banerjee, S. R., Babich, J. W. & Zubieta, J. (2006). *Inorg. Chim. Acta*, **359**, 1603–1612.

[bb4] Banerjee, S. R., Murali, K. L., Lazarova, N., Wei, L., Valliant, J. F., Stephenson, K. A., Babich, J. W., Maresca, K. P. & Zubieta, J. (2002). *Inorg. Chem.* **41**, 6417–6425. 10.1021/ic020476e 12444786

[bb5] Banerjee, S. R. & Zubieta, J. (2005). *Acta Cryst.* C**61**, m275–m277. 10.1107/S0108270105009820 15930663

[bb6] Brandenburg, K. & Putz, H. (2005). *DIAMOND* Crystal Impact GbR, Bonn, Germany.

[bb7] Bruker (2008). *APEX2*, *SAINT* and *SADABS* Bruker AXS Inc., Madison, Wisconsin, USA.

[bb8] Farrugia, L. J. (1999). *J. Appl. Cryst.* **32**, 837–838.

[bb9] Kunz, P. C., Bruckmann, N. E. & Spingler, B. (2007). *Eur. J. Inorg. Chem.* **3**, 394–399.

[bb10] Moore, A. L., Bucar, A.-K., MacGillivray, L. R. & Benny, P. D. (2010). *Dalton Trans.* **39**, 1926–1928. 10.1039/b921413e 20148205

[bb11] Raszeja, L., Maghnouj, A., Hahn, S. & Metzler-Nolte, N. (2011). *ChemBioChem*, **12**, 371–376. 10.1002/cbic.201000576 21290535

[bb12] Sheldrick, G. M. (2008). *Acta Cryst.* A**64**, 112–122. 10.1107/S0108767307043930 18156677

[bb13] Wei, L., Babich, J. W., Ouellette, W. & Zubieta, J. (2006). *Inorg. Chem.* **45**, 3057–3066. 10.1021/ic0517319 16562962

